# Propensity Score-Matched Analysis to Identify Pathways Associated with Loss of Sodium Iodide Symporter in Papillary Thyroid Cancer

**DOI:** 10.3390/cimb44040101

**Published:** 2022-03-26

**Authors:** Fang Lee, Chi-Yu Kuo, Chung-Hsin Tsai, Shih-Ping Cheng

**Affiliations:** 1 Department of Surgery, MacKay Memorial Hospital, Mackay Medical College, Taipei 104215, Taiwan; leefang.4828@mmh.org.tw (F.L.); kiwikuo.4289@mmh.org.tw (C.-Y.K.); cst@mmh.org.tw (C.-H.T.); 2 Institute of Biomedical Sciences, Mackay Medical College, New Taipei City 252005, Taiwan; 3 Department of Pharmacology, School of Medicine, College of Medicine, Taipei Medical University, Taipei 110301, Taiwan

**Keywords:** sodium iodide symporter, thyroid cancer, propensity score matching, gene set enrichment analysis

## Abstract

Sodium iodide symporter (NIS) expression in thyroid follicular cells plays an important role in normal physiology and radioactive iodine therapy for thyroid cancer. Loss of NIS expression is often seen in thyroid cancers and may lead to radioiodine refractoriness. To explore novel mechanisms of NIS repression beyond oncogenic drivers, clinical and RNA-seq data from the thyroid cancer dataset of The Cancer Genome Atlas were analyzed. Propensity score matching was used to control for various genetic background factors. We found that tumoral NIS expression was negatively correlated with tumor size. Additionally, low NIS expression was the only factor associated with recurrence-free survival in a Cox multivariate regression analysis. After matching for clinicopathologic profiles and driver mutations, the principal component analysis revealed distinct gene expressions between the high and low NIS groups. Gene set enrichment analysis suggested the downregulation of hedgehog signaling, immune networks, and cell adhesions. Positively enriched pathways included DNA replication, nucleotide excision repair, MYC, and Wnt/β-catenin pathways. In summary, we identified several potential targets which could be exploited to rescue the loss of NIS expression and develop redifferentiation strategies to facilitate radioactive iodine therapy for thyroid cancer.

## 1. Introduction

Sodium iodide symporter (NIS), encoded by the SLC5A5 gene on chromosome 19p13.11, is a transmembrane glycoprotein that mediates active transport of iodide from the bloodstream into thyroid follicular cells [1]. NIS-mediated radioactive iodine (RAI) uptake is the cornerstone of the diagnosis and treatment of thyroid cancer with RAI. For patients with differentiated thyroid cancer, RAI therapy is associated with a lower risk of all-cause and cancer-specific mortality [2]. When thyroid cancer becomes RAI-refractory, the 10-year survival rate of patients with thyroid cancer drops to less than 20%, and the life expectancy decreases to 3 to 5 years [3].

Impaired RAI uptake or accumulation accounts for the refractoriness of RAI therapy. This parallels the dedifferentiation process during tumor progression, with a decrease in, or loss of, NIS expression and/or targeting to the plasma membrane [4]. Several oncogenic pathways are associated with the loss of NIS expression [5]. The most well-known molecular link to NIS loss in thyroid cancer is the BRAF V600E mutation—the most common genetic alteration in papillary thyroid cancer [6]. A selective BRAF inhibitor, dabrafenib, has been shown to stimulate RAI uptake in clinical trials [7]. However, novel therapeutic targets are still urgently needed to tailor NIS redifferentiation and further improve patient outcomes.

In the present study, we used clinical and molecular data from the dataset of The Cancer Genome Atlas for Thyroid Cancer (TCGA-THCA) to explore potential pathways associated with the loss of NIS expression. To eliminate the confounding effects of different genetic backgrounds, a propensity score-matched approach was adopted to provide a balanced and innovative comparison.

## 2. Materials and Methods

### 2.1. Data Acquisition

Clinicopathologic information, Mutation Annotation Format files, and normalized level 3 RNA-seq data were downloaded from the Genomic Data Commons Data Portal (https://portal.gdc.cancer.gov/; accessed on 11 June 2021). The American Joint Committee on Cancer (AJCC) stage, American Thyroid Association (ATA) recurrence risk group, recurrence events, and patients’ vital status were obtained and updated. Gene expression levels were acquired at RNA-seq by expectation maximization (RSEM) values [8]. The thyroid differentiation score (TDS) and BRAF-RAS score (BRS) were referenced from the initial TCGA-THCA global analysis [9]. A lower or negative BRS represents more mitogen-activated protein kinase (MAPK) output by oncogenic BRAF V600E. Additionally, the BRAF-RAS class was based on the gene expression profile, which could be classified as BRAF-like (resembling those with the BRAF V600E mutation) or RAS-like (resembling those with RAS mutations).

A formal power analysis was not conducted. Clinicopathologic parameters were compared between groups using the chi-square test, Fisher’s exact test, or the Cochran–Armitage trend test for categorical variables and the Mann–Whitney U test for continuous variables. Correlations between parameters were investigated using Spearman’s rank correlation coefficients. Survival curves were generated using the Kaplan–Meier method and compared using the log-rank statistic [10]. Variables with *p* < 0.1 in univariate analysis were subjected to multivariate Cox regression modeling.

### 2.2. Propensity Score Matching

Propensity score matching was performed to adjust for differences in baseline covariates and driver mutations that might have a major impact on gene expression profiling. A 1:1 matching scheme was utilized with the nearest-neighbor matching method without a replacement. The propensity score was assessed using logistic regression, which included clinicopathologic profiles (age, sex, female, tumor subtype, tumor size, multifocality, extrathyroidal extension, lymph node metastasis, and stage) and driver mutations (BRAF, RAS, and TERT promoter mutations) [11]. Balance in covariate distribution between groups after the matching process was examined by post-matching analysis.

### 2.3. Principal Component Analysis

Principal component analysis was performed to visually explore the distribution pattern of different groups according to their gene expression profiles [12]. The percent explained variance was examined using eigenvalue decomposition. The first two components providing an adequate representation of the data were used to produce the biplot.

### 2.4. Pathway Analysis

To prevent the problem of arbitrary cutoff thresholds in over-representation analysis, gene set enrichment analysis (GSEA) was performed [13]. The GSEA strategy requires considering entire genes in the enrichment analysis without selecting significant genes. The enrichment score was calculated from the rank order of all genes in the annotation category. The Molecular Signatures Database v7.5.1 was retrieved from the GSEA website (https://www.gsea-msigdb.org/; accessed on 7 February 2022). The Kyoto Encyclopedia of Genes and Genomes (KEGG) and Pathway Interaction Database (PID) databases were examined to identify potential pathways associated with NIS loss.

## 3. Results

**Figure 1:**
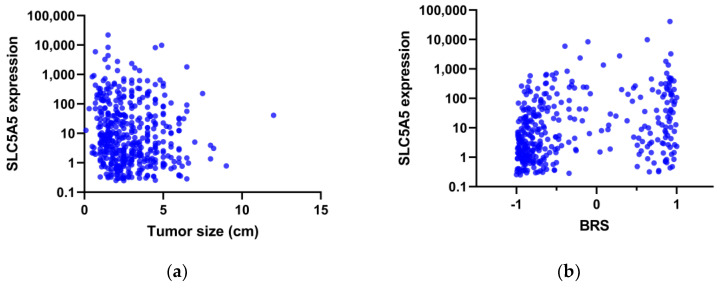
Scatter plots of the expression of SLC5A5 (sodium iodide symporter) versus (**a**) tumor size and (**b**) BRAF-RAS scores (BRS) in the TCGA-THCA dataset.

A total of 500 patients with papillary thyroid cancer were included in the analysis before matching. As expected, the NIS expression level was closely correlated with TDS (Spearman’s rho = 0.542, *p* < 0.001). The age at diagnosis was not associated with the NIS expression level (*p* = 0.728), but there was a negative correlation between the tumor size and NIS expression (Spearman’s rho = −0.153, *p* = 0.001, Figure 1a).

Data are expressed as the number (percentage) or median (interquartile range).

After a median split, patients were assigned to either the high or low NIS groups. Patients in the low NIS group had larger tumor size, more advanced extrathyroidal extension, and a higher risk of recurrence (Table 1). A higher prevalence of the BRAF V600E mutation was seen in the low NIS group, consistent with a positive correlation between BRS and NIS expression (Spearman’s rho = 0.303, *p* < 0.001, Figure 1b). Of interest, TERT promoter mutations were more frequent in the low NIS group than in the high NIS group.

No difference in overall survival was observed between the groups (log-rank *p* = 0.172). However, patients in the low NIS group had a significantly shorter recurrence-free survival time (*p* < 0.001). Extrathyroidal extension, lymph node metastasis, AJCC stage, TERT promoter mutation, and NIS expression status were associated with recurrence-free survival in univariate analysis (Table 2). In multivariate analysis, low NIS expression was the only factor associated with recurrence-free survival, with a hazard ratio of 3.136 (*p* = 0.014).

**Figure 2:**
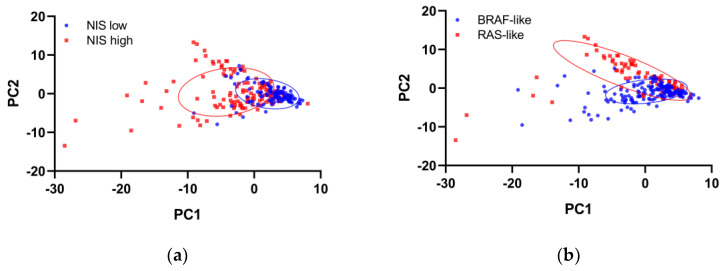
Principal component analysis plots of the gene expression profiling of the propensity score-matched TCGA-THCA dataset, grouped by the expression of (**a**) the sodium iodide symporter (NIS) or (**b**) the BRAF-RAS class.

Following matching for tumor size, AJCC stage, and driver mutations, 121 patients in each group were selected for transcriptome analyses. The groups after matching showed good balance in clinicopathologic features (Supplementary Table S1). Principal component analysis of transcriptome profiling showed distinct gene expression between the high and low NIS groups, although BRAF-like and RAS-like classes remained separable (Figure 2).

**Figure 3:**
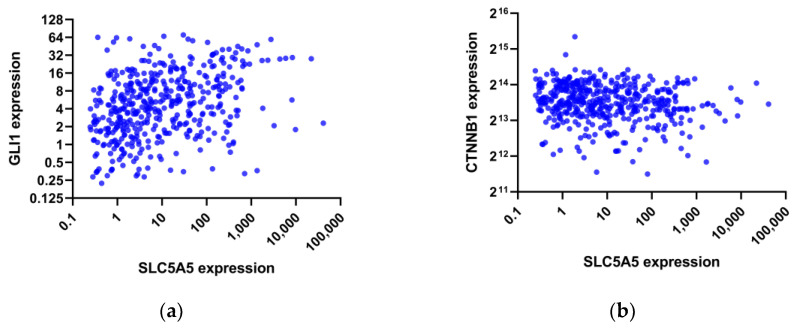
Scatter plots of the expression of SLC5A5 (sodium iodide symporter) versus (**a**) GLI1 expression and (**b**) CTNNB1 expression in the TCGA-THCA dataset.

Pathway analysis was performed by GSEA to compare gene expression between the two groups. KEGG pathways enriched in the low NIS group included downregulation of hedgehog signaling, immune networks, and cell adhesions, while nucleotide excision repair and DNA replication were upregulated (Table 3). Consistently, negatively enriched PID pathways were the ones primarily relevant to T-cell immunity (Table 4). In contrast, MYC, LKB1, and Wnt/β-catenin pathways were positively enriched in the low NIS group. To further corroborate these findings, we analyzed the association between the expression of NIS and key effectors in the entire TCGA-THCA patient cohort. As shown in Figure 3, NIS expression was positively correlated with GLI1 (Spearman’s rho = 0.386, *p* < 0.001) and was negatively correlated with CTNNB1 (Spearman’s rho = −0.138, *p* = 0.002).

## 4. Discussion

Refractoriness to RAI therapy represents an unmet therapeutic need in thyroid cancer. Early identification of patients who are refractory to RAI therapy not only saves patients from ineffective radiation exposure but also expedites the implementation of alternative treatment plans. An updated meta-analysis reported that extrathyroidal extension, BRAF V600E mutation, TERT promoter mutations, and high-risk histological subtypes were associated with RAI refractoriness [14]. In this study, we substantiated that the loss of NIS expression was associated with high-risk clinicopathologic features of papillary thyroid cancer. Consistent with a previous report [15], we found that NIS expression was inversely correlated with tumor size. This finding suggests that the NIS loss occurs during the process of tumor progression, and molecular pathways that are in the relatively late stages probably play some role in NIS downregulation. The recognition of molecular mechanisms that are involved in the loss of NIS expression and RAI refractoriness may shed some light on the puzzles in our understanding of thyroid dedifferentiation processes.

Age is an important prognostic factor in thyroid cancer, and thyroid cancer is the only cancer type that incorporates age in the tumor-node-metastasis stage. The thyroid tissue of children has smaller follicles and higher expression of NIS, pendrin, and dual oxidases [16]. Additionally, younger patients were more likely to have RAI-avid distant metastases [17]. It was therefore reasoned that physiological and pathological changes associated with aging can influence NIS expression and RAI responsiveness. However, we found that tumor NIS expression was unrelated to patient age. Our results are in agreement with a previous study from Portuguese researchers [18]. The negative impact of aging on prognosis likely results from metabolic alterations and immune dysregulation [19].

We previously highlighted that the low expression of thyroid differentiation genes was associated with disease recurrence [20]. In the present study, low NIS expression was identified as a predictor of recurrence-free survival, independent of oncogenic drivers. MAPK and PI3K/AKT pathways are well-characterized mechanisms leading to NIS repression [5]. BRAF mutations are particularly enriched in RAI-refractory metastases [21]. Without adequately matching, the results of comparisons would be substantially confounded by these classic pathways. The strength of our approach lies in balancing the genetic discrepancy between the high and low NIS groups.

In the low NIS group, GSEA indicated the downregulation of hedgehog signaling. Although experimental evidence suggests that the hedgehog pathway promotes the self-renewal of thyroid cancer stem cells [22], hedgehog pathway activity is generally reduced in papillary thyroid cancer [23]. We recently reported that ethacridine, a TAZ activator, induced differentiation, despite TAZ being conventionally considered to preserve the stemness properties of progenitor cells [24]. The blockage of hedgehog signaling by cyclopamine could result in markedly decreased type 3 deiodinase expression [25]. Whether a cause-and-effect relationship exists between hedgehog signaling and NIS downregulation requires further investigation.

The Wnt/β-catenin pathway is also essential for stem cell maintenance. A previous study showed that β-catenin overexpression decreased cytomembrane localization of NIS and RAI uptake ability in FTC-133 cells [26]. However, in rat thyroid follicular cell lines, β-catenin as downstream of thyrotropin and insulin-like growth factor 1 increased the expression of Pax8 and NIS [27]. It should be noted that differential β-catenin expression between benign and malignant thyroid neoplasms was seen in cytoplasmic or nuclear localization (indicating accumulation of the Wnt canonical pathway) but not membranous localization (non-Wnt signaling) [28]. Furthermore, cytoplasmic/nuclear β-catenin expression was strongly associated with galectin-3 expression. Galectin-3 is a carbohydrate-binding protein commonly overexpressed in thyroid cancer and can serve as a diagnostic biomarker of thyroid cancer. Recently, we reported that galectin-3 inhibitors could suppress anoikis resistance and invasive capacity in thyroid cancer cells [29]. Activation of the Wnt/β-catenin pathway along with NIS downregulation is a fascinating topic for future work.

A non-pump function of intracellular non-membranous NIS may have a tumorigenic role [30]. In the current study, we observed upregulation of the DNA replication pathway and MYC pathway in the low NIS group. Network analysis revealed that MYC is one of the essential proteins that regulate NIS expression in anaplastic thyroid cancer [31]. Interestingly, a BRD4 inhibitor, JQ1, was able to suppress MYC and enhance NIS expression [32]. MYC-stimulated cell proliferation may potentiate genotoxic stress induced by oncogenic drivers. NIS repression can also result from DNA damage involving ATM-mediated mechanisms [33]. We noted that dermatopontin is among the top-ranked differentially expressed genes among both the high and low NIS groups. Dermatopontin impeded the proliferation of thyroid cancer cells through MYC repression [34]. It will be intriguing to determine whether MYC transcriptionally regulates NIS expression.

Taken together, through a propensity score-matched analysis, our transcriptome analysis identified several novel pathways that could serve as potential targets in future studies to reverse the loss of NIS expression in thyroid cancer. More research is warranted to determine the translatability of these findings for developing redifferentiation strategies for the management of patients with RAI-refractory thyroid cancer.

## Figures and Tables

**Table 1:** Clinicopathologic features of the TCGA-THCA patient cohort, grouped according to the expression of sodium iodide symporter (NIS) by median split.

	Low NIS (*n* = 250)	High NIS (*n* = 250)	*p* Value
Age (years)	46 (35–58)	46 (35–58)	0.971
Female	180 (72%)	185 (74%)	0.614
Subtype			0.281
Classic	179 (72%)	175 (70%)	
Follicular variant	48 (19%)	55 (22%)	
Tall cell variant	21 (8%)	14 (6%)	
Other variants	2 (1%)	6 (2%)	
Tumor size (cm) ^a^	2.7 (2.0–4.0)	2.3 (1.5–3.7)	0.002
Multifocality ^a^	104 (42%)	122 (50%)	0.072
Extrathyroidal extension ^a,b^			0.001
None	149 (62%)	181 (75%)	
Minimal (T3)	78 (32%)	55 (23%)	
Advanced (T4)	15 (6%)	4 (2%)	
Lymph node metastasis	122 (49%)	102 (41%)	0.072
Stage ^b^			0.058
Stage I	131 (52%)	149 (60%)	
Stage II	27 (11%)	22 (9%)	
Stage III	52 (21%)	57 (23%)	
Stage IV	40 (16%)	22 (9%)	
Risk of recurrence ^a,b^			0.001
Low	74 (32%)	97 (44%)	
Intermediate	136 (59%)	121 (55%)	
High	20 (9%)	4 (2%)	
BRAF V600E mutation ^a^	146 (59%)	88 (36%)	<0.001
RAS mutation ^a^	23 (9%)	29 (12%)	0.387
TERT promoter mutation ^a^	28 (14%)	11 (6%)	0.006

^a^ Missing data excluded. ^b^ Cochran–Armitage trend test.

**Table 2:** Cox regression analysis of recurrence-free survival in the TCGA-THCA patient cohort.

	Univariate			Multivariate		
	Hazard Ratio	95% CI	*p* Value	Hazard Ratio	95% CI	*p* Value
Age at diagnosis	1.012	0.994–1.031	0.180			
Tumor size	1.171	1.000–1.372	0.050	0.999	0.806–1.237	0.989
Multifocality	1.090	0.606–1.961	0.774			
Extrathyroidal extension	1.817	1.010–3.266	0.046	0.796	0.360–1.758	0.572
Lymph node metastasis	1.917	1.060–3.466	0.031	1.339	0.909–1.972	0.139
AJCC stage	1.443	1.135–1.834	0.003	1.344	0.954–1.895	0.091
BRAF V600E mutation	1.450	0.797–2.638	0.223			
RAS mutation	1.223	0.482–3.104	0.672			
TERT promoter mutation	2.806	1.217–6.471	0.015	1.611	0.643–4.033	0.309
Low NIS expression	3.610	1.791–7.276	<0.001	3.136	1.255–7.832	0.014

**Table 3:** Top 20 Kyoto Encyclopedia of Genes and Genomes (KEGG) pathways associated with expression loss of the sodium iodide symporter.

Pathway	Enrichment Score	Adjusted *p*
KEGG_HEDGEHOG_SIGNALING_PATHWAY	−0.547	0.001
KEGG_INTESTINAL_IMMUNE_NETWORK_FOR_IGA_PRODUCTION	−0.760	0.004
KEGG_PRIMARY_IMMUNODEFICIENCY	−0.773	0.006
KEGG_ASTHMA	−0.753	0.010
KEGG_CYTOKINE_CYTOKINE_RECEPTOR_INTERACTION	−0.513	0.011
KEGG_HEMATOPOIETIC_CELL_LINEAGE	−0.601	0.019
KEGG_CHEMOKINE_SIGNALING_PATHWAY	−0.494	0.021
KEGG_T_CELL_RECEPTOR_SIGNALING_PATHWAY	−0.484	0.021
KEGG_CELL_ADHESION_MOLECULES_CAMS	−0.533	0.022
KEGG_B_CELL_RECEPTOR_SIGNALING_PATHWAY	−0.493	0.024
KEGG_COMPLEMENT_AND_COAGULATION_CASCADES	−0.498	0.031
KEGG_GLYCOSAMINOGLYCAN_BIOSYNTHESIS_CHONDROITIN_SULFATE	−0.570	0.031
KEGG_RIBOFLAVIN_METABOLISM	+0.542	0.035
KEGG_ONE_CARBON_POOL_BY_FOLATE	+0.580	0.037
KEGG_TYPE_I_DIABETES_MELLITUS	−0.690	0.040
KEGG_AUTOIMMUNE_THYROID_DISEASE	−0.630	0.041
KEGG_GRAFT_VERSUS_HOST_DISEASE	−0.734	0.047
KEGG_NUCLEOTIDE_EXCISION_REPAIR	+0.458	0.047
KEGG_ALLOGRAFT_REJECTION	−0.747	0.048
KEGG_DNA_REPLICATION	+0.624	0.048

**Table 4:** Top 20 Pathway Interaction Database (PID) pathways associated with expression loss of the sodium iodide symporter.

Pathway	Enrichment Score	Adjusted *p*
PID_TCR_CALCIUM_PATHWAY	−0.668	0.001
PID_MYC_PATHWAY	+0.521	0.004
PID_CD8_TCR_PATHWAY	−0.644	0.018
PID_IL12_STAT4_PATHWAY	−0.676	0.018
PID_ALPHA_SYNUCLEIN_PATHWAY	−0.454	0.027
PID_PI3KCI_PATHWAY	−0.524	0.027
PID_LKB1_PATHWAY	+0.397	0.029
PID_HIV_NEF_PATHWAY	−0.557	0.031
PID_FRA_PATHWAY	−0.574	0.031
PID_IL23_PATHWAY	−0.659	0.032
PID_CD8_TCR_DOWNSTREAM_PATHWAY	−0.523	0.034
PID_TCR_PATHWAY	−0.616	0.038
PID_WNT_CANONICAL_PATHWAY	+0.519	0.047
PID_BETA_CATENIN_DEG_PATHWAY	+0.502	0.048
PID_ILK_PATHWAY	−0.420	0.050
PID_CD40_PATHWAY	−0.493	0.053
PID_NFAT_TFPATHWAY	−0.510	0.053
PID_AP1_PATHWAY	−0.452	0.056
PID_ERBB_NETWORK_PATHWAY	+0.572	0.056
PID_THROMBIN_PAR4_PATHWAY	−0.577	0.062

## Data Availability

All data generated or analyzed during this study are included in this published article and its Supplementary Materials files.

## Supplementary Materials

The following supporting information can be downloaded at https://www.mdpi.com/article/10.3390/cimb44040101/s1, Table S1: Clinicopathologic features of the TCGA-THCA patient cohort, grouped by the expression of sodium iodide symporter (NIS) after propensity score matching.
