# Supplementary material for: Propensity Score-Matched Analysis to Identify Pathways Associated with Loss of Sodium Iodide Symporter in Papillary Thyroid Cancer
Source: Curr Issues Mol Biol. 2022 Mar 26;44(4):1488–96. doi: 10.3390/cimb44040101 (PMC9164071; doi:10.3390/cimb44040101)
Supplement: Supplementary file 1 [file cimb-44-00101-s001.zip › cimb-1608562-supplementary.pdf]

Supplementary Table S1 Clinicopathologic features of the TCGA-THCA patient cohort grouped by the expression of sodium-iodide symporter (NIS) after propensity score matching

	**NIS low (n = 121)**	**NIS high (n = 121)**	***p* value**
Age (years)	47 (35-58)	46 (35-56)	0.934
Female	87 (72%)	87 (72%)	1.000
Subtype			0.884
Classic	79 (65%)	82 (68%)	
Follicular variant	33 (27%)	29 (24%)	
Tall cell variant	8 (7%)	8 (7%)	
Other variants	1 (1%)	2 (2%)	
Tumor size (cm)	2.5 (1.7-3.2)	2.1 (1.5-3.4)	0.348
Multifocality [a]	52 (44%)	52 (44%)	0.908
Extrathyroidal extension [a,b]			0.946
None	92 (79%)	94 (79%)	
Minimal (T3)	24 (21%)	24 (20%)	
Advanced (T4)	1 (1%)	1 (1%)	
Lymph node metastasis	37 (31%)	38 (31%)	0.889
Stage [b]			0.274
Stage I	68 (56%)	76 (63%)	
Stage II	18 (15%)	16 (13%)	
Stage III	25 (21%)	22 (18%)	
Stage IV	10 (8%)	7 (6%)	
Risk of recurrence [a,b]			0.895
Low	58 (51%)	58 (52%)	
Intermediate	54 (48%)	54 (48%)	
High	1 (1%)	0 (0%)	
BRAF V600E mutation	72 (60%)	72 (60%)	1.000
RAS mutation	17 (14%)	14 (12%)	0.564
TERT promoter mutation	8 (7%)	8 (7%)	1.000

[a] Missing data excluded. [b] Cochran-Armitage trend test.

Data are expressed as the number (percentage) or median (interquartile range).
